# p53 Plays an Important Role in Cell Fate Determination after Exposure to Microcystin-LR

**DOI:** 10.1289/ehp.1001899

**Published:** 2010-04-26

**Authors:** Shota Takumi, Masaharu Komatsu, Tatsuhiko Furukawa, Ryuji Ikeda, Tomoyuki Sumizawa, Hitomi Akenaga, Yuta Maeda, Kohji Aoyama, Koji Arizono, Seiichi Ando, Toru Takeuchi

**Affiliations:** 1 Department of Environmental Medicine, Graduate School of Medical and Dental Sciences, Kagoshima University, Kagoshima, Japan; 2 Environmental and Symbiotic Sciences, Prefectural University of Kumamoto, Kumamoto, Japan; 3 Department of Food and Chemical Biology, Faculty of Fisheries, Kagoshima University, Kagoshima, Japan; 4 Department of Molecular Oncology and; 5 Department of Clinical Pharmacy and Pharmacology, Graduate School of Medical and Dental Sciences, Kagoshima University, Kagoshima, Japan; 6 Department of Environmental Toxicology, Institute of Industrial Ecological Sciences, University of Occupational and Environmental Health, Kitakyushu, Japan

**Keywords:** Akt, apoptosis, β-catenin, cyclin D1, GSK-3β, microcystin-LR, OATP1B3, p53, proliferation, siah-1

## Abstract

**Background:**

Microcystin-LR, a cyclic heptapeptide, possesses the ability to inhibit the serine/threonine protein phosphatases PP1 and PP2A and, consequently, exhibits acute hepatocytotoxicity. Moreover, microcystin-LR induces cellular proliferation, resulting in tumor-promoting activity in hepatocytes. However, mechanisms that regulate the balance between cell death and proliferation after microcystin-LR treatment remain unclear.

**Objective:**

We examined the contribution of the transcription factor p53, as well as that of the hepatic uptake transporter for microcystin-LR, organic anion transporting polypeptide 1B3 (OATP1B3), to the cellular response to microcystin-LR exposure.

**Methods:**

We analyzed intracellular signaling responses to microcystin-LR by immunoblotting and real-time reverse-transcriptase polymerase chain reaction techniques using HEK293 human embryonic kidney cells stably transfected with *SLCO1B3* (HEK293-OATP1B3). In addition, we analyzed the effect of attenuation of p53 function, via the p53 inhibitor pifithrin-α, and knockdown of *p53* mRNA on the cytotoxicity of microcystin-LR using a 3-(4,5-dimethylthiazol-2-yl)-2,5-diphenyltetrazolium bromide (MTT) assay.

**Results:**

Microcystin-LR induced the phosphorylation and accumulation of p53 in HEK293-OATP1B3 cells, which resulted in up-regulation of the expression of p53 transcript targets, including *p21* and seven in absentia homolog 1 (*siah-1*). In addition, microcystin-LR activated Akt signaling through the phosphorylation of Akt and glycogen synthase kinase 3β. Although Akt signaling was activated, the accumulation of p53 led cells to apoptosis after treatment with 50 nM microcystin-LR for 24 hr. Both pharmacological inhibition of transcription factor activity of p53 by pifithrin-α and knockdown of p53 with small hairpin RNA attenuated the susceptibility of HEK293-OATP1B3 cells to microcystin-LR.

**Conclusions:**

This study demonstrates the importance of p53 in the regulation of cell fate after exposure to microcystin-LR. Our results suggest that, under conditions of p53 inactivation (including *p53* mutation), chronic exposure to low doses of microcystin-LR may lead to cell proliferation through activation of Akt signaling. Results of this study may contribute to the development of chemoprevention and chemotherapeutic approaches to microcystin-LR poisoning.

Microcystin-LR is a cyclic peptide released by several bloom-forming toxic cyanobacteria (Carmichael and Falconer 1993). Understanding the toxicity of microcystin-LR is of paramount importance because of both the potency of its acute cytotoxicity and its tumor-promoting activity in hepatocytes of animals and humans (Dietrich and Hoeger 2005; Gehringer 2004). Microcystin-LR toxicity is primarily caused by inhibition of the serine/threonine protein phosphatases PP1 and PP2A, thereby influencing the regulation of the balance between cellular protein phosphorylation and dephosphorylation (Dietrich and Hoeger 2005; Gehringer 2004). Several PP1 and PP2A inhibitors, such as okadaic acid, nodularin, and microcystin-LR, are classified as tumor promoters (Fujiki and Suganuma 1999). Several epidemiological studies have indicated that consumption of cyanobacteria-contaminated water can favor the growth of human hepatocellular carcinoma (Falconer and Humpage 2005; Ueno et al. 1996; Yuan et al. 2006). Microcystin-LR has poor cell membrane permeability except for the membranes of hepatocytes (Chong et al. 2000; Eriksson et al. 1990; Nong et al. 2007; Runnegar et al. 1995). Recently, we (Komatsu et al. 2007) and others (Fischer et al. 2005; Monks et al. 2007) reported that the expression of the human hepatocyte uptake transporter OATP1B1 or OATP1B3 was critical for the selective uptake of microcystin-LR into hepatocytes and for induction of its fatal cytotoxicity. However, the intracellular regulatory mechanisms that determine whether a cell will die or survive in response to microcystin-LR have not been clarified.

The molecular basis of the tumor-promoting ability of microcystin-LR is unclear but most likely involves protein phosphatase inhibition leading to hyperphosphorylation of many cellular proteins and, consequently, destruction of cell-cycle control (Fujiki and Suganuma 1999; Guy et al. 1992; Messner et al. 2006). Several signaling pathways have been implicated in cellular effects of microcystin-LR. Previous studies have demonstrated that microcystin-LR can stabilize *Xenopus* and zebrafish β-catenin protein levels by suppressing glycogen synthase kinase-3β (GSK-3β), a serine/threonine protein kinase that phosphorylates β-catenin, resulting in its proteasomal degradation (Li et al. 2001; Wang et al. 2005). β-Catenin is a multifunctional protein that plays an important role in the transduction of wingless int (Wnt) signals, which contributes to hyperplasia and tumorigenic progression, and in cellular adhesion by linking the cytoplasmic domains of cadherin to each other (Grimes and Jope 2001; Olmeda et al. 2003; Orford et al. 1999; Wang et al. 2005). In general, a low cytoplasmic level of β-catenin is maintained through interaction with a protein complex consisting of adenomatous polyposis coli, Axin, PP2A, and GSK-3β (Ding et al. 2000). Recently, p53 has been reported to induce proteasomal degradation of β-catenin through the transactivation of seven in absentia homolog 1 (*siah-1*), which encodes an E3 ubiquitin ligase (Jang et al. 2005; Jung et al. 2007; Kim et al. 2004; Park et al. 2006). Accumulation of β-catenin can induce the expression of various genes, such as *cyclin D1* and *c-myc*, which promote cell proliferation and transformation of many cell types (Seki et al. 2006). Phosphorylation of GSK-3β at Ser^9^, which results in an inactive form of GSK-3β, is mediated by activated Akt, which is a serine/threonine protein kinase that is a well-established antiapoptotic protein (Frame and Cohen 2001; Grimes and Jope 2001). Akt regulates many cellular processes implicated in tumorigenesis (Altomare and Testa 2005; Lu et al. 2007). Recently, constitutive activation of Akt was reported in microcystin-transformed immortalized colorectal crypt cells (Zhu et al. 2005). We also recently reported that microcystin-LR induces apoptosis through activation of mitogen-activated protein kinase (MAPK) (Komatsu et al. 2007). Moreover, there is increasing evidence of a functional relationship between p53 and MAPK. Thus, MAPK that is phosphorylated after exposure to cellular stress then phosphorylates p53 and, consequently, induces apoptosis (Guan et al. 2008; Karunakaran et al. 2008; Liu et al. 2009; Shimada et al. 2003).

p53 functions as a transcription factor that, in response to a variety of cellular stresses, including DNA damage and hypoxia, regulates its downstream genes and p53-mediated pathways in an attempt to repair DNA damage through induction of cell-cycle arrest. p53 is involved in G_1_ and G_2_ cell-cycle arrest through activation of the p21 cyclin-dependent kinase inhibitor (Burns and El-Deiry 1999).

In the present study, we used wild-type p53-expressing HEK293 human embryonic kidney cells stably transfected with OATP1B3, to demonstrate that a lethal concentration of microcystin-LR stimulated not only cellular signal transduction, leading to cell death, but also cell survival signals. These data suggest that if cell damage inflicted by microcystin-LR cannot be repaired, then p53 probably promotes cell apoptosis. However, if p53 is inactivated, chronic exposure to a low dose of microcystin-LR might lead to cell proliferation through activation of Akt signaling.

## Materials and Methods

### Reagents and antibodies

Reagents and antibodies used are described in the Supplemental Material (doi:10.1289/ehp.1001899).

### Cell culture

Wild-type HEK293 cells were stably transfected with the plasmids pcDNA3.1-SLCO1B3 (HEK293-OATP1B3 cells) or pcDNA3.1-control vector (HEK293-CV cells), which were generated previously (König et al. 2000; Letschert et al. 2004). Cells were cultured in Minimum Essential Medium (MEM; Sigma Chemical Co., St. Louis, MO, USA) supplemented with 10% fetal calf serum (FCS), 100 U/mL penicillin, 100 μg/mL streptomycin (MEM/10% FCS) and 400 μg/mL G418 at 37°C, 100% humidity, and 5% CO_2_.

### Preparation of nuclear fractions

Cells were resuspended in buffer A (10 mM Tris-Cl, pH 8.0, 10 mM KCl, 1.5 mM MgCl_2_, 60 μM phenylmethylsulfonyl fluoride, and a proteinase inhibitor cocktail) at 4°C, incubated for 20 min on ice, and homogenized for 30 sec. The homogenate was centrifuged at 3,000 × *g* for 5 min, and the postnuclear supernatant was clarified by centrifugation for 30 min at 15,000 × *g*. The first pellet was washed twice with buffer A and resuspended in buffer A containing 1% Nonidet P-40, and this suspension was homogenized for 30 sec. Homogenates were collected by centrifugation for 30 min at 15,000 × *g*, and supernatants were collected as the nuclear fraction. Prepared nuclear fractions were analyzed by immunoblotting as previously described (Komatsu et al. 2000, 2007). For details of immunoblotting, see Supplemental Material (doi:10.1289/ehp.1001899).

### Immunoprecipitation

After incubation with microcystin-LR, cells were lysed with Lysis buffer (10 mM Tris-HCl, pH 8.0, 10 mM KCl, 1 mM EDTA, 1% Nonidet P-40, 1% protein inhibitor cocktail,and 1 mM NaF). Following homogenization, the lysates were centrifuged at 10,000 × *g* for 30 min at 4°C. One milliliter of cell lysate was incubated overnight at 4°C with 5 μL of agarose-conjugated anti-p53 antibody. The pellet was washed four times with Lysis buffer and then suspended in SDS-polyacrylamide gel Laemmli sample buffer. After SDS/PAGE and immunoblotting with the respective phospho-p53 antibodies, phosphorylation of p53 at Ser^6^, Ser^9^, Ser^15^, Ser^20^, Ser^37^, Ser^46^, and Ser^392^ was analyzed in the same samples. After stripping with stripping buffer (0.5 M Tris-Cl, pH 6.8 containing 1% 2-ME) for 30 min at 50˚C, the blots were reprobed with an anti-p53 antibody.

### Detection of ubiquitination

HEK293-OATP1B3 cells were treated with 50 nM microcystin-LR for 12 hr under serum-free conditions. The cells were treated with 10 μM lactacystin for 2 hr before cell harvest to inhibit proteasomal degradation of β-catenin. Whole-cell lysates from harvested cells were then analyzed by immunoblot analysis.

### Real-time reverse-transcriptase polymerase chain reaction (RT-PCR)

Total cellular RNA was extracted from HEK293-OATP1B3 cells using TRIzol (Invitrogen, Carlsbad, CA, USA) according to the manufacturer’s instructions. cDNA was synthesized by reverse transcription of total RNA using reverse transcriptase (Toyobo, Osaka, Japan) and an oligo(dT)20 primer (Toyobo). The resulting cDNA was amplified using the following three PCR steps: preincubation at 95°C for 10 min, 45 cycles of denaturation at 95°C for 15 sec and then annealing at 56°C for 30 sec, and finally extension at 72°C for 30 sec, using FastStart Universal SYBR Green Master (Roche, Basel, Switzerland). The fluorescent signal from the samples was acquired at the end of the elongation step. Real-time PCR was performed using the Thermal Cycler Dice Real Time System (Takara, Otsu, Japan). The following sense and antisense primers, respectively, were used for PCR: *siah-1*, 5′-GACTGGCACAACTGCATCCA-3′ and 5′-AGCCAAGTGCGAATGGATC-3′; *cyclin D1*, 5′-AATGACCCCGCACGATT-3′ and 5′-GCACAGAGGGCAACGAAGG-3′; *c-myc*, 5′-TGCTGCCAAGAGGGTCAAG-3′ and 5′-GCCTCCAAGACGTTGTGAGT-3′; glyceraldehyde 3-phosphate dehydrogenase (*GAPDH*), 5′-TGGACCTGACCTGCCGTCTA-3′ and 5′-CCCTGTTGCTGTAGCCAAATTC-3′. For each sample, the relative expression level was calculated using cycle time (Ct) values, which were normalized against *GAPDH*. Relative quantification (fold change) between different samples was then determined according to the 2^−ΔΔCt^ method (Livak and Schmittgen 2001).

### Pifithrin-α treatment

HEK293-OATP1B3 cells in 170 μL MEM were pretreated without (6% DMSO vehicle control) or with 10 μL pifithrin-α (30 μM) for 5 hr and were then incubated for 3 days with 20 μL of microcystin-LR. After incubation, microcystin-LR cytotoxicity was analyzed by analysis of cell survival using the 3-(4,5-dimethylthiazol-2-yl)-2,5-diphenyltetrazolium bromide (MTT) assay [see Supplemental Material (doi:10.1289/ehp.1001899) for details].

### Knockdown of p53 mRNA

HEK293-OATP1B3 cells were transiently transfected with the small hairpin RNA (shRNA) plasmid pSuppressorNeo p53 using Lipofectamine 2000 (Invitrogen) according to the manufacturer’s protocol to generate *p53* small interfering RNA (siRNA). Cells were then incubated for 72 hr. To determine *p53* expression by immunoblotting, 4 × 10^6^ cells in 10 mL MEM/10% FCS were inoculated into 100-mm dishes. After 24 hr, the cells were harvested and the cell lysates were analyzed. For MTT analysis, exponentially growing transfected HEK293-OATP1B3 cells were trypsinized and harvested, and equal numbers of cells (1.6 × 10^4^) in 180 μL MEM/10% FCS were then inoculated into each well of a 96-well microplate and assayed using the MTT assay.

### Statistical analysis

Differences between groups were analyzed using Wilcoxon–Mann–Whitney test. *p*-Values < 0.05 were considered significant.

## Results

### Activation of the p53 pathway by microcystin-LR

**Figure 1:**
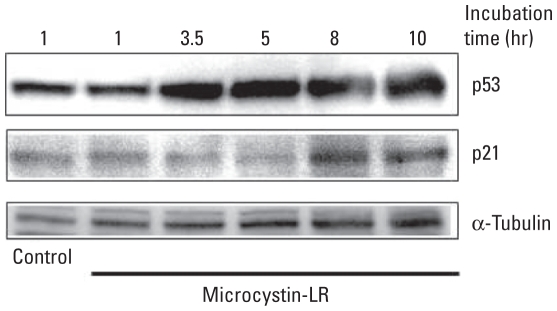
Microcystin-LR treatment of HEK293-OATP1B3 cells activates the p53 signaling pathway. HEK293-OATP1B3 cells were incubated without (control) or with 50 nM microcystin-LR for the indicated times. Cell lysates were then immunoblotted for p53; for protein product of its transcriptional target, gene *p21*; or for α-tubulin, which was used as an internal control. Results are representative of three independent experiments.

**Figure 2:**
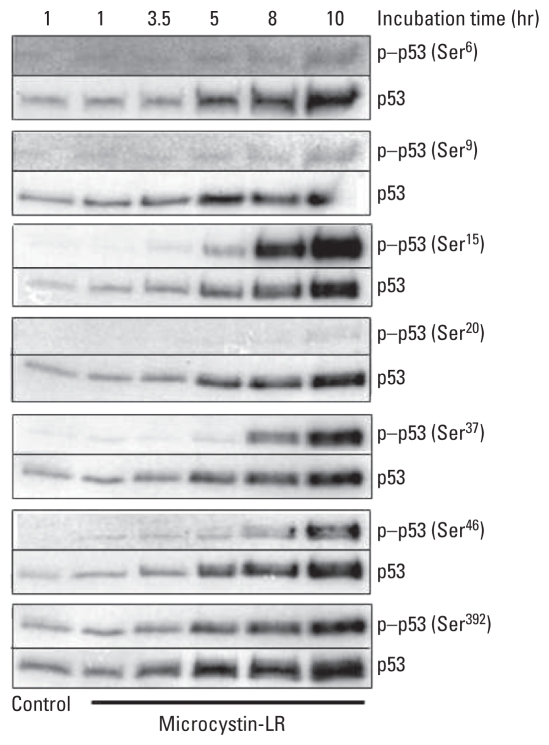
Site-selective phosphorylation of p53 by microcystin-LR cell treatment. Cell lysates of HEK293-OATP1B3 cells treated with 50 nM microcystin-LR for the indicated times were immunoprecipitated with agarose-conjugated anti-p53 antibodies. The pellet was analyzed by immunoblotting using antibodies against specific phosphorylated sites in p53 (p-p53), and the blots were reprobed with an anti-p53 phosphorylation site–independent antibody. Results are representative of three independent experiments.

HEK293-OATP1B3, but not HEK293-CV, cells were sensitive to microcystin-LR [see Supplemental Material, Figure 1 (doi:10.1289/ehp.1001899)]. To determine if p53 is involved in microcystin-LR–induced apoptosis, we examined p53 protein stability and its phosphorylation in HEK293-OATP1B3 cells by immunoblot analysis (Figures 1 and 2). The level of p53 protein increased after 3.5 hr of exposure to a lethal concentration (50 nM) of microcystin-LR (Figure 1). After accumulation of p53, the expression level of protein product of its transcriptional target gene *p21* also increased (Figure 1). After 3.5–5 hr, we observed phosphorylation of p53 at Ser^15^, which reduces the ability of p53 to bind to its negative regulator, the oncoprotein MDM2, and at Ser^392^, which is increased in human tumors. In both cases phosphorylation coincided with the accumulation of p53 protein (Figure 2). After these early phosphorylation events, we observed delayed phosphorylation of p53 at Ser^37^, which impairs the ability of MDM2 to bind p53, thus promoting both the accumulation and activation of p53 in response to DNA damage, and at Ser^46^, which is important in regulating the ability of p53 to induce apoptosis. Phosphorylation was slightly detectable at these sites after 3.5 and 5 hr but was considerably stronger after 8–10 hr of exposure to 50 nM microcystin-LR (Figure 2). In addition, we observed weak phosphorylation of p53 at Ser^6^ and Ser^9^, which are mediated by the casein kinases CK1δ and CK1ɛ, and at Ser^20^, which is induced by DNA damage and leads to reduced interaction between p53 and MDM2, as reflected by accumulation of p53 protein (Figure 2).

### Activation of the Akt pathway by microcystin-LR

**Figure 3:**
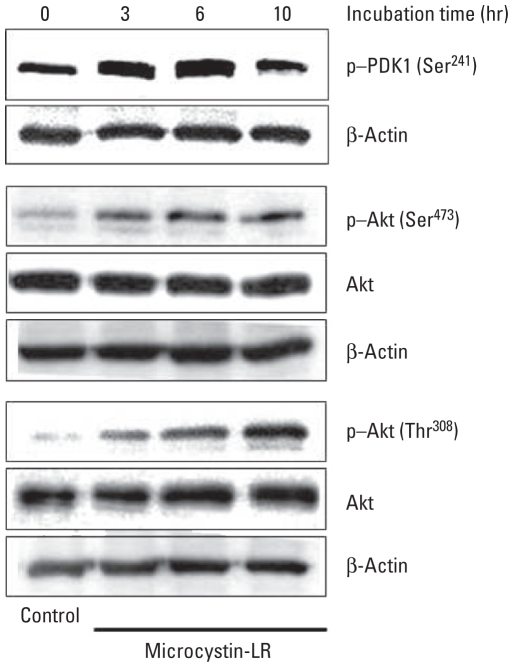
Microcystin-LR activates the Akt signaling pathway in HEK293-OATP1B3 cells. Cell lysates of HEK293-OATP1B3 cells treated with 50 nM microcystin-LR for the indicated times were analyzed by immunoblotting using anti-phosphorylated PDK1 (p-PDK1) and anti-phosphorylated phosphAkt (p-Akt) antibodies. The blots were reprobed with antibodies against the Akt protein; β-actin was used as an internal control. Results are representative of three independent experiments.

Substrates of PP2A, a target of microcystin-LR, include components of the well-described Akt cell survival signaling pathway. We therefore analyzed the effect of microcystin-LR on the activation of such components, including 3-phosphoinositide–dependent protein kinase 1 (PDK1), Akt, and GSK-3β. The intensity of PDK1 phosphorylation at Ser^241^, which correlates with PDK1 activity, was slightly and transiently increased 3–6 hr after exposure to microcystin-LR and recovered to control levels after 10 hr. In contrast, phosphorylation of Akt at the activating phosphorylation sites Ser^473^ and Thr^308^ was considerably enhanced by exposure of the cells to 50 nM microcystin-LR. However, the expression level of Akt in HEK293-OATP1B3 cells was not changed by treatment with 50 nM microcystin-LR for up to 10 hr (Figure 3).

**Figure 4:**
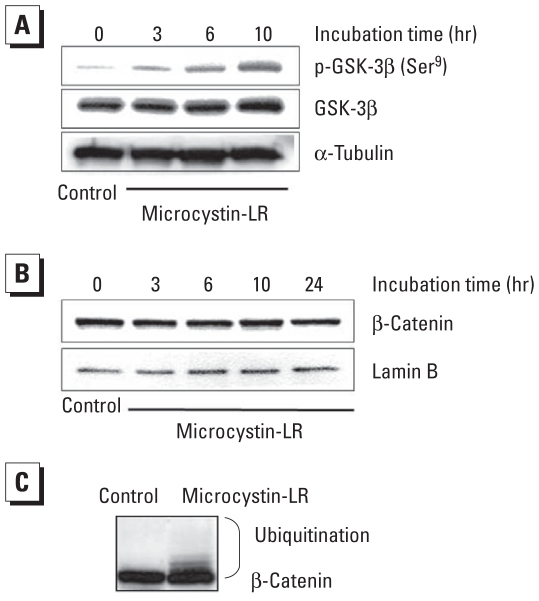
Correlation between phosphorylation of GSK-3β and nuclear accumulation of β-catenin in HEK293-OATP1B3 cells after treatment with 50 nM microcystin-LR. (*A*) Immunoblot analysis of phosphorylated (p-)GSK-3β (Ser^9^). Blots were reprobed with an anti-GSK-3β antibody; α-tubulin was used as an internal control. (*B*) Immunoblot analysis of β-catenin accumulated in the nuclear fraction of HEK293-OATP1B3 cells. The blots were reprobed with an antibody against lamin B, which was used as an internal control for the nuclear fraction. (*C*) Immunoblot analysis of β-catenin in whole HEK293-OATP1B3 cells analyzed by immunoblot analysis. The results are representative of three independent experiments.

In HEK293-OATP1B3 cells, phosphorylation of GSK-3β increased in a time- dependent manner after incubation with 50 nM microcystin-LR for up to 10 hr. In contrast, the expression level of GSK-3β did not change (Figure 4A). These data suggest that microcystin-LR indirectly regulates the activity of both Akt and GSK-3β.

### Correlation of β-catenin with microcystin-LR cytotoxicity

We next determined if microcystin-LR–induced phosphorylation of GSK-3β led to an enhanced nuclear translocation of its substrate protein β-catenin. Surprisingly, the level of β-catenin in the nuclear fraction of cells after up to 24 hr incubation with 50 nM microcystin-LR indicated that β-catenin levels in the nucleus were equivalent before and after treatment (Figure 4B). In addition, we detected ubiquitination of β-catenin in the presence of 10 μM proteasome inhibitor lactacystin after treatment with microcystin-LR (Figure 4C).

### Analysis of cyclin D1 and c-myc mRNA expression

**Figure 5:**
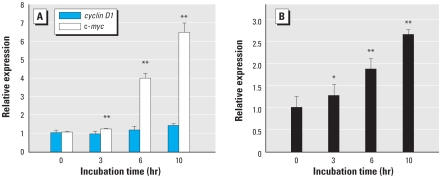
Effect of 50 nM microcystin-LR on the expression of *cyclin D1*, *c-myc*, and *siah-1* mRNA in HEK293-OATP1B3 cells. mRNA levels of *cyclin D1* and c*-myc* (*A*) and of *siah-1* (*B*) were quantified by real-time RT-PCR, and the mRNA expression level of each sample was calculated relative to that of *GAPDH*, which was used as an internal control. The data represent mean ± SD of three independent experiments, each performed in triplicate. *p < 0.05, and **p < 0.01, compared with control before incubation with microcystin-LR.

To confirm that β-catenin levels in the nucleus were not increased after microcystin-LR treatment, we analyzed the mRNA levels of the β-catenin targets *cyclin D1* and *c-myc* after treatment with 50 nM microcystin-LR. The mRNA level of *cyclin D1* was not changed after microcystin-LR treatment, but, unexpectedly, that of *c-myc* was considerably up-regulated (Figure 5A), suggesting that microcystin-LR activated *c-myc* through signaling pathways other than β-catenin.

### Induction of siah-1 by microcystin-LR

The lack of an increase in nuclear levels of β-catenin despite the fact that GSK-3β was phosphorylated after microcystin-LR treatment suggested that β-catenin might be ubiquitinated and degraded through another pathway that is activated by microcystin-LR. β-Catenin can be degraded after p53-dependent up-regulation of the E3 ubiquitin ligase gene *siah-1* and its protein product. We therefore determined whether *siah-1* mRNA levels were modulated after treatment of cells with 50 nM microcystin-LR. Real-time RT-PCR analysis indicated that microcystin-LR treatment led to the up-regulation of *siah-1* mRNA (Figure 5B), suggesting that siah-1–mediated degradation of β-catenin may be responsible for the lack of nuclear accumulation of β-catenin.

### Effects of p53 on the cytotoxicity of microcystin-LR

**Figure 6:**
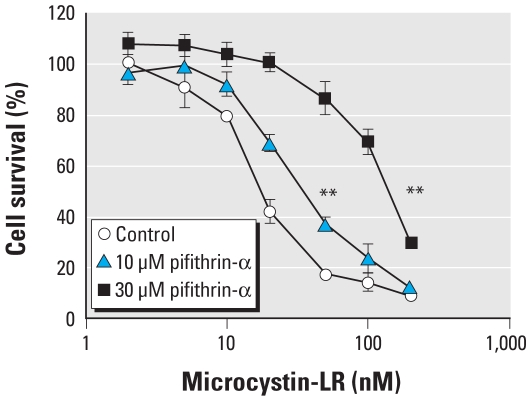
Effect of inhibition of transcription factor activity of p53 by pifithrin-α on cell viability. Data represent mean ± SD of three independent experiments, each performed in triplicate. **p < 0.01, compared with control without pifithrin-α treatment.

**Figure 7:**
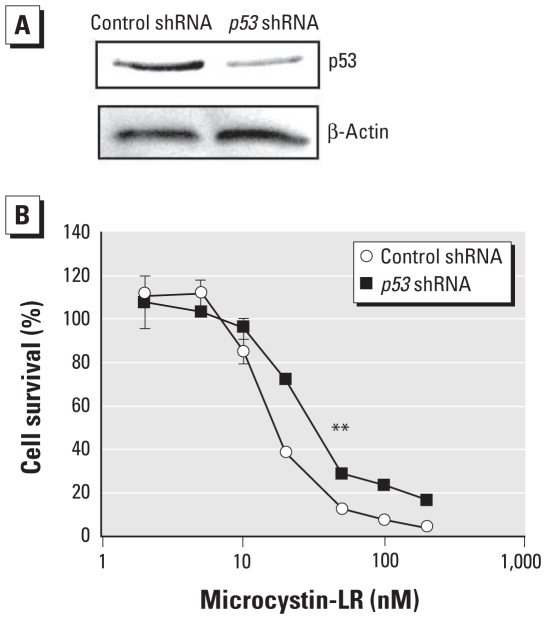
Knockdown of *p53* mRNA attenuated the susceptibility of HEK293-OATP1B3 cells to microcystin-LR cytotoxicity. Cells were transfected with the pSuppressorNeo p53 plasmid or the pSuppressorNeo control plasmid and incubated for 72 hr. (*A*) Immunoblot analysis of cells for *p53* expression using β-actin as a loading control; results are representative of three independent experiments. (*B*) Viability of cells treated with microcystin-LR for an additional 3 days and then analyzed using the MTT assay. Data represent mean ± SD of triplicate samples in each group. **p < 0.01 compared with control cells transfected with control shRNA plasmid.

To verify whether p53 was critically important for microcystin-LR–induced apoptosis, we analyzed the effect of pretreatment with an inhibitor of the transcription factor activity of p53, pifithrin-α, for 5 hr on the cytotoxicity of microcystin-LR in HEK293-OATP1B3 cells. Pifithrin-α concentration-dependently attenuated the susceptibility of HEK293-OATP1B3 cells to microcystin-LR compared with vehicle (6%DMSO)-treated (control) cells (Figure 6). The median inhibitory concentrations (IC_50_) were approximately 2 and 8 times higher in cells that were pretreated with 10 μM and 30 μM pifithrin-α, respectively, than in controls. We further analyzed the acute cytotoxicity of microcystin-LR in HEK293-OATP1B3 cells after knockdown of *p53* following transfection with a *p53* shRNA plasmid. This plasmid effectively decreased the expression level of p53 protein (Figure 7A). The susceptibility of HEK293-OATP1B3 cells to microcystin-LR was attenuated to a greater extent by knockdown of *p53* than by transfection of cells with a plasmid encoding a scrambled shRNA sequence, which does not show significant homology to rat, mouse, or human gene sequences (Figure 7B).

## Discussion

Microcystin-LR is of great interest because of its acute cytotoxicity and its tumor-promoting activity in hepatocytes of animals and humans (Gehringer 2004; Li et al. 2009; Wang et al. 2005). In our previous studies aimed at clarifying the onset of liver-specific toxicity of microcystin-LR (Komatsu et al. 2007; Nong et al. 2007), we demonstrated that transfection of OATP1B1 (*SLCO1B1*) or OATP1B3 (*SLCO1B3*) cDNA into HEK293 cells increased their sensitivity to, and their accumulation of, microcystin-LR and, consequently, induced apoptosis in those cells. The use of these cell lines permits exclusive analysis of the effects of low concentrations of microcystin-LR on the cellular balance between phosphatase and kinase activity, which is a crucial regulatory mechanism for cellular homeostasis. PP2A is one of the key proteins in this regulatory mechanism (Junttila et al. 2008; Lin et al. 2007; Yan et al. 1997) and is known to be a target molecule of microcystin-LR (Gehringer 2004). However, because of the poor membrane permeability of microcystin-LR, a complete understanding of the effect of low concentrations of microcystin-LR on various cell signaling pathways remains unclear. Here, we used HEK293-OATP1B3 cells to clarify the molecular basis of the balance between cell death and cell survival after microcystin-LR treatment. In this study, we focused on the effects of low concentrations of microcystin-LR on the phosphorylation of p53 and on Akt signaling pathways.

In our previous study using HEK293-OATP1B3 cells, microcystin-LR activated MAPKs, including ERK1/2 (extracellular signal-regulated kinase 1/2), JNK (c-Jun N-terminal kinase), and p38, through inhibition of PP2A (Komatsu et al. 2007). In the present study, we demonstrated that once MAPKs are activated by microcystin-LR, they activate downstream kinases that phosphorylate a number of substrates, including p53, which plays a central role in the cellular response to various stresses (Wu 2004). Treatment with 50 nM microcystin-LR for 10 hr induced phosphorylation of p53 at Ser^15^, Ser^37^, Ser^46^, and Ser^392^. In contrast, Ser^6^, Ser^9^, and Ser^20^ were weakly phosphorylated (Figure 2). The site at which p53 is phosphorylated is dependent on the kind of stress applied or the cell type assayed (Kurihara et al. 2007; Wu 2004). Phosphorylation of p53 at Ser^15^ promotes p53 stabilization by attenuating the interaction between p53 and its negative regulator MDM2. The resulting accumulation of phosphorylated p53 resulted in increased expression of p21 and *siah-1* (Figures 1 and 5B). The *p21* gene is a transcriptional target of p53 and induces inactivation of the cyclin-dependent kinase complex CDK2/cyclin E that controls the initiation of DNA synthesis (Hegarat et al. 2005). Therefore, elevated levels of p53 and p21 after treatment with microystin-LR are probably responsible for induction of cell-cycle arrest. These results are consistent with our previously reported flow cytometric analysis, which revealed that the G_1_ phase, and the sub-G_1_ proportion of HEK293-OATP1B3 cells, increased after exposure to 50 nM microcystin-LR for 24 hr (Komatsu et al. 2007). Furthermore, the phosphorylation of p53 at Ser^46^ correlates well with the induction of apoptosis (Smith et al. 2003; Yoshida et al. 2006). Therefore, these events ultimately induce apoptosis of HEK293-OATP1B3 cells after exposure to microcystin-LR.

Recently, crosstalk between p53 and Akt has been hypothesized to play a critical role in the regulation of cell fate determination (Oren 2003), and the Akt signaling pathway plays an important role in promoting cell proliferation (Lu et al. 2007). Zhu et al. (2005) reported that transformation of immortalized colorectal crypt cells by microcystin is involved in the constitutive activation of Akt and MAPK. In the present study using HEK293-OATP1B3 cells, phosphorylation of Akt at Ser^473^ was enhanced after 3 hr incubation with 50 nM microcystin-LR (Figure 3). Subsequently, phosphorylation of Akt at Thr^308^ was gradually enhanced to reach a high level of phosphorylation (Figure 3). Therefore, microcystin-LR treatment may activate Akt to phosphorylate its downstream targets, such as GSK-3β kinase (Frame and Cohen 2001). Indeed, microcystin-LR induced the phosphorylation of GSK-3β at Ser^9^ (Figure 4A), resulting in a loss of GSK-3β kinase activity. Phosphorylation of Akt is regulated by the balance between the activities of PP2A and PDK1, which are upstream of Akt activation (Ivaska et al. 2002; Li et al. 2003). However, analysis of the phosphorylation level of PDK1 indicated that it was slightly and transiently increased and subsequently recovered to control levels after treatment with microcystin-LR for 10 hr, suggesting that PDK1 may not play an important role in microcystin-LR-activation of Akt (Figure 3). In a previous study using unstimulated HEK293 cells, Casamayor et al. (1999) found that PDK1 was strongly phosphorylated at Ser^241^, which is critical for PDK1 activity, and that the level of PDK1 phosphorylation at this site was unaffected by stimulation with insulin-like growth factor-1. Hence, the phosphorylation of Akt observed in the present study is likely due to PP2A inhibition by microcystin-LR.

Because microcystin-LR can activate Akt to phosphorylate and thereby inhibit GSK-3β, we next analyzed the effect of incubation of HEK293-OATP1B3 cells with 50 nM microcystin-LR on the translocation of the GSK-3β substrate β-catenin from the cytoplasm to the nucleus. Inhibition of GSK-3β-mediated phosphorylation of β-catenin plays a role in the translocation of β-catenin to the nucleus (Ding et al. 2000; Grimes and Jope 2001). We therefore predicted that β-catenin might accumulate in the nucleus of HEK293-OATP1B3 cells after incubation with 50 nM microcystin-LR. However, under these conditions, nuclear accumulation of β-catenin was negligible (Figure 4B). In agreement with this finding, the transcription of *cyclin D1*—one of the transcriptional targets of β-catenin and a key regulator of cell-cycle progression (Alt et al. 2000; Diehl et al. 1998; Seki et al. 2006)—was not enhanced by microcystin-LR (Figure 5A). We further analyzed the effect of microcystin-LR on the mRNA level of *c-myc*, which is also a well-known transcriptional target of β-catenin. In contrast to the lack of effect of microcystin-LR on *cyclin D1* expression levels, microcystin-LR strongly up-regulated *c-myc* mRNA (Figure 5A). A significant increase of *c-myc* may play a role in cellular accumulation of p53 (Matsumura et al. 2003). However, the *c-myc* promoter is targeted not only by β-catenin but also by other transcription factors under the control of multiple signal transduction cascades, including the MAPK cascade (Vervoorts et al. 2006). Therefore, analysis of the mechanism by which *c-myc* transcription is enhanced remains to be investigated.

Nuclear accumulation of β-catenin is highly related to tumor promotion (Cui et al. 2001, 2003), and PP2A activity also correlates with β-catenin degradation (Bos et al. 2006; Li et al. 2001). The nuclear dephosphorylated β-catenin level is decreased after treatment with okadaic acid compared with control without okadaic acid (Bordonaro et al. 2007). To resolve the lack of correlation between the inactivation of GSK-3β and the cellular β-catenin level after exposure to microcystin-LR, we considered the possibility that β-catenin might be degraded via the p53–siah-1 pathway after microcystin-LR treatment. Indeed, we detected ubiquitination of β-catenin after treatment with microcystin-LR (Figure 4C). According to previous studies, stabilization of p53 can induce GSK-3β–independent proteasomal degradation of β-catenin through the transactivation of the E3 ubiquitin ligase *siah-1*, which is a transcriptional target gene of p53 (Jang et al. 2005; Jung et al. 2007; Kim et al. 2004; Park et al. 2006). Real-time RT-PCR analysis indicated that *siah-1* mRNA significantly increased in a time-dependent manner after exposure to microcystin-LR and that this increase coincided with the accumulation of p53 (Figure 5B). These results suggest that the p53–siah-1 pathway may play an important role in preventing microcystin-LR–induced β-catenin accumulation and may therefore block cell-cycle progression in HEK293-OATP1B3 cells. In this scenario, if p53 were to be inactivated by, for example, a mutagen such as aflatoxin B1 (Szymańska et al. 2009) or by oxidative stress produced by microcystin-LR itself (Nong et al. 2007), chronic exposure to low-dose microcystin-LR might lead to cell proliferation through activation of Akt signaling.

To verify that p53 is critically related to microcystin-LR–induced apoptosis, we analyzed the cytotoxicity of microcystin-LR in HEK293-OATP1B3 cells in which p53 was inhibited or down-regulated. Both pharmacological inhibition of transcription factor activity of p53 (Figure 6) and knockdown of p53 with shRNA (Figure 7) attenuated the susceptibility of HEK293-OATP1B3 cells to microcystin-LR, suggesting that p53, at least in part, is associated with microcystin-LR–induced apoptosis. p53 promotes apoptosis mediated by the mitochondrial pathway (Green and Kroemer 2009). However, caspase-3 is not involved in the apoptosis that occurs after exposure to microcystin-LR (Ding et al. 2002; Komatsu et al. 2007). Further studies are needed to clarify the contribution of p53 to cell fate determination after exposure of HEK293-OATP1B3 cells to microcystin-LR.

## Conclusion

**Figure 8:**
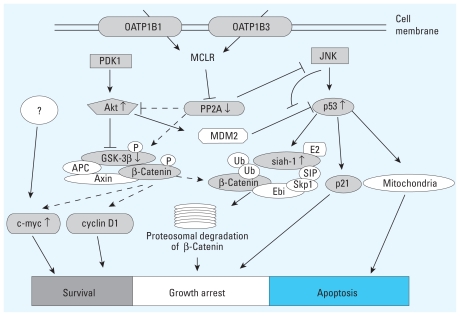
Model of the p53-Akt cross-regulatory network that mediates the cellular response to microcystin-LR stress in HEK293-OATP1B3 cells. Microcystin-LR simultaneously induces activation of both Akt and p53 signaling. However, p53 signaling is more active than Akt signaling in HEK293-OATP1B3 cells after treatment with 50 nM microcystin-LR. As a result, p53 induces arrest of the cell cycle and apoptosis by the induction of several genes, including *p21* and *siah-1*. Abbreviations: P, phosphorylation; Ub, ubiquitination. Dashed lines indicate proposed pathways; small arrows, transport; large arrows, activation; blunt-end lines (⊥), inhibition. Up (↑) and down (↓) arrows adjacent to protein names indicate activation and inactivation, respectively. Proteins shaded gray were analyzed in the present study and/or in our previous studies (Komatsu et al. 2007).

The present study has demonstrated that HEK293-OATP1B3 cells are a useful tool for analysis of the cellular effects of low concentration of microcystin-LR. We revealed that the fate of HEK293-OATP1B3 cells is determined through activation of various cell signaling pathways, including MAPK pathways, especially the JNK pathway (Komatsu et al. 2007), as well as through Akt and p53 signaling pathways. We have also shown the importance of p53 in microcystin-LR–induced apoptosis (Figure 8). Our results suggest that conditions of inactivated p53, coupled with chronic exposure to low-dose microcystin-LR, may lead to cell proliferation through activation of Akt signaling. Results of this study may contribute to the development of chemoprevention and chemotherapeutic approaches to microcystin-LR poisoning.
